# Nanotechnological approaches for diagnosis and treatment of ovarian cancer: a review of recent trends

**DOI:** 10.1080/10717544.2022.2132032

**Published:** 2022-10-19

**Authors:** Haigang Ding, Juan Zhang, Feng Zhang, Yan Xu, Wenqing Liang, Yijun Yu

**Affiliations:** a Department of Gynecology, Shaoxing Maternity and Child Health Care Hospital, Shaoxing, China; b Obstetrics and Gynecology Hospital, Shaoxing University, Shaoxing, China; c Intensive Care Unit, Zhoushan Hospital of Traditional Chinese Medicine Affiliated to Zhejiang Chinese Medical University, Zhoushan, China; d Medical Research Center, Zhoushan Hospital of Traditional Chinese Medicine Affiliated to Zhejiang Chinese Medical University, Zhoushan, China

**Keywords:** Ovarian cancer, nanotechnology, drug nanocarriers, diagnosis and treatment

## Abstract

Formulations from nanotechnology platform promote therapeutic drug delivery and offer various advantages such as biocompatibility, non-inflammatory effects, high therapeutic output, biodegradability, non-toxicity, and biocompatibility in comparison with free drug delivery. Due to inherent shortcomings of conventional drug delivery to cancerous tissues, alternative nanotechnological-based approaches have been developed for such ailments. Ovarian cancer is the leading gynecological cancer with higher mortality rates due to its reoccurrence and late diagnosis. In recent years, the field of medical nanotechnology has witnessed significant progress in addressing existing problems and improving the diagnosis and therapy of various diseases including cancer. Nevertheless, the literature and current reviews on nanotechnology are mainly focused on its applications in other cancers or diseases. In this review, we focused on the nanoscale drug delivery systems for ovarian cancer targeted therapy and diagnosis, and different nanocarriers systems including dendrimers, nanoparticles, liposomes, nanocapsules, and nanomicelles for ovarian cancer have been discussed. In comparison to non-functionalized counterparts of nanoformulations, the therapeutic potential and preferential targeting of ovarian cancer through ligand functionalized nanoformulations’ development has been reviewed. Furthermore, numerous biomarkers such as prostatic, mucin 1, CA-125, apoptosis repeat baculoviral inhibitor-5, human epididymis protein-4, and e-cadherin have been identified and elucidated in this review for the assessment of ovarian cancer. Nanomaterial biosensor-based tumor markers and their various types for ovarian cancer diagnosis are explained in this article. In association, different nanocarrier approaches for the ovarian cancer therapy have also been underpinned. To ensure ovarian cancer control and efficient detection, there is an urgent need for faster and less costly medical tools in the arena of oncology.

## 1. Introduction

**Figure 1.**
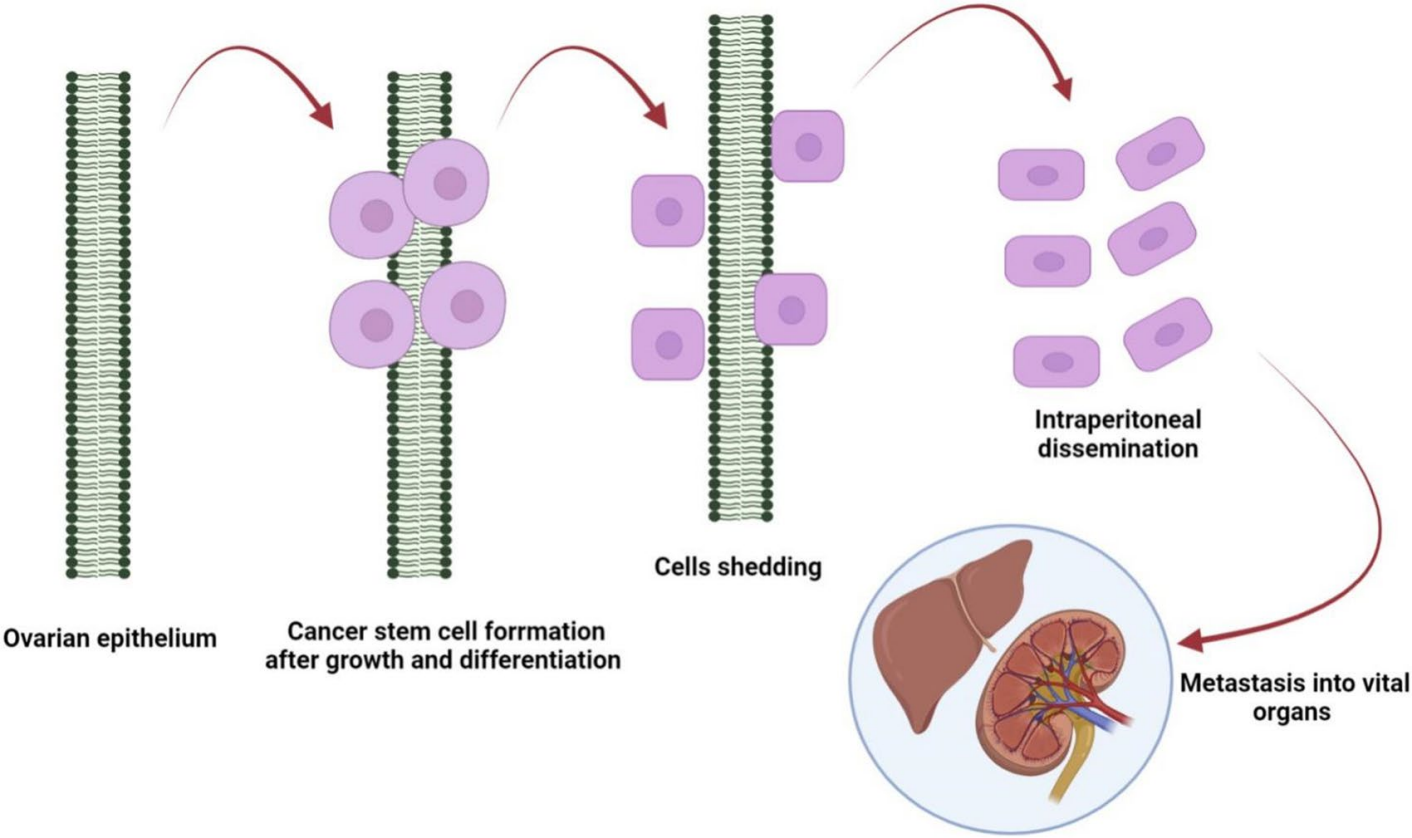
Various steps involved in the carcinogenesis and metastasis of ovarian cancer.

Among gynecological malignancies, ovarian cancer manifests higher mortality rates that is attributed to its reoccurrence and late diagnosis (Bhatt et al., 2016; Rojas et al., 2016). Within omentum and ovary, the ovarian cancer is characterized by intraperitoneal metastasis and diffuse nature malignant ascites (Chen et al., 2019; Stewart et al., 2019). Patients with ovarian cancer (75%) initially show certain intra-abdominal ailments that support ovarian carcinoma diagnosis and stage III ovarian cancer patients (<40%) have shown a survival rate of approximately 5 years (Giampaolino et al., 2019). Those patients suffering from ovarian cancer relapse have shown a limit up to peritoneum since during therapy the use of intraperitoneal route has shown many toxicities in such patients. As per available literature data, the cross-talk between conventional chemotherapeutics and ovarian cancer cell is not friendly that has led to resistance offered by cancer cells towards these therapeutic cargoes. Consequently, medication resistance and recurrence have been observed in ovarian cancer cells (Tarhriz et al., 2019). The process and steps involved in the ovary carcinogenesis are depicted in Figure 1.

Stem cell formation takes place on the ovarian surface due to proliferation and differentiation of ovarian surface epithelium. Then extracellular matrix gets degrade and the dissociation of cells takes place from primary cancer. The dissociated cells become abdominal free-floating cells or clumps together that forms multicellular aggregates.

In the context of patient survival, chemotherapy and radiation therapy is under clinical trials to evaluate the ovarian cancer initial stage and approach the benefits of survival (Boevé et al., 2019). In patients with advanced stages of ovarian cancer, surgery >1 cm in diameter is considered as first-line treatment followed by cisplatin/paclitaxel-based administration via intraperitoneal or intravenous route. In this regard, a gynecology-oncology group conducted clinical trials and compared cisplatin administration via intraperitoneal and intravenous routes. Results showed that intraperitoneal administration mimicked the intravenous administration of cisplatin (Bai et al., 2019; Ghafouri-Fard et al., 2022). Generally, a high relapse of ovarian cancer was observed along with poor treatment results in response to all applied treatment strategies. It indicates that to develop better and life-saving therapeutic regimens, more efforts are required to cope with ovarian cancer patients (Abolhasani Zadeh et al., 2022). The major reason for high death rate due to ovarian cancer is attributed to high rate and worse consequences of proliferation within the abdominal cavity of ovarian cancer patients as well as late diagnosis (Bozkurt et al., 2016; Gurashi et al., 2018). Indeed, in the early detection and diagnosis of ovarian cancer, technology advancement is need of the day in this field that helps us to rely on physical examination, clinical histories, physical diagnosis, ultrasound evaluation and CA-125 serum protein detection (Peres et al., 2019). Interestingly, in ovarian cancer women (>80%) have shown an elevated level of CA-125 serum protein. In contrast, sometimes the elevated level of CA-125 serum protein poses ineffective treatment instead of being effective diagnosis determinant that could be considered during therapy (Chang et al., 2019). In addition, in women with ovarian cancer, Lysophosphatidic acid is another diagnostic marker and as compared to CA-125 serum protein it has found more effective diagnostic marker at early stages of ovarian cancer (Chae et al., 2022).

**Figure 2.**
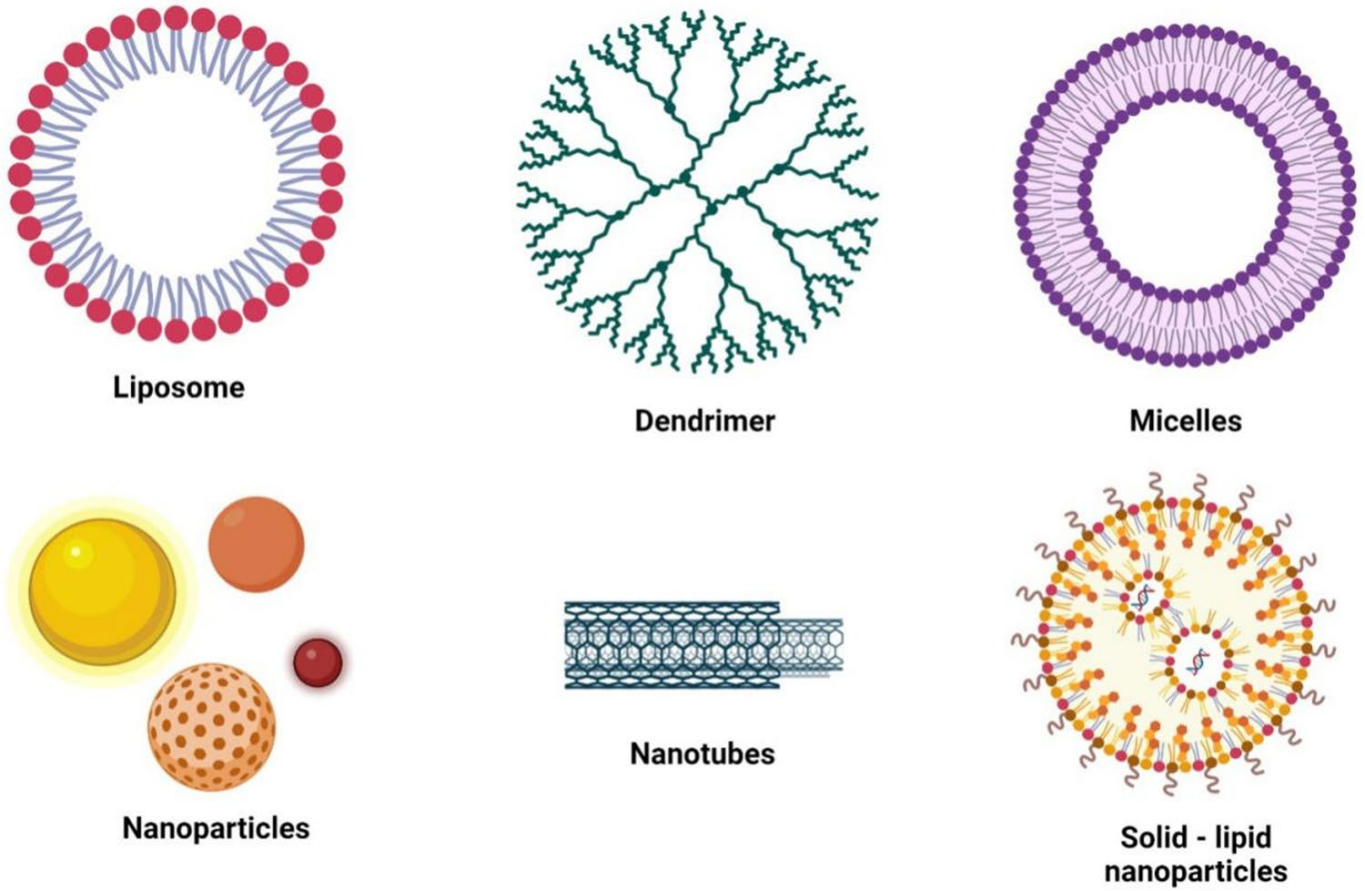
Depiction of few nanocarriers used in the diagnosis and treatment/therapy.

In the early phase ovarian cancer detection, the current treatment and diagnostic methods are not enough efficient and sensitive as well. Furthermore, delayed diagnosis has been observed as a virtue of non-specified detection and high costs of the applied methods. Recently, nanotechnology is emerged as a growing field in the treatment and diagnosis of ovarian cancer that offers the development of novel and potential strategies to overcome ovarian cancer (Zhou et al., 2022). The targeted delivery of cancer therapeutic drugs, hydrophobic drugs and stabilization of carriers have been achieved through the application of nanocarriers along with overcoming the issue to systemic toxicity (Salari et al., 2021). Moreover, various fluorescent and contrast agents were used as nanocarriers for diagnosis purposes that efficiently delivered the targeting moieties (Kotcherlakota et al., 2017). Some nanocarriers were used as nano theranostics due to their inherent optical properties and their ability of cell destruction due to conversion into high-state energy within the cells. In this regard, in vivo and in vitro studies were conducted in ovarian cancer mice models applying a combination of quantum dots for diagnosis and imaging purpose (Baghbani & Moztarzadeh, 2017; Yao et al., 2018). A few of the diagnostic and treatment/theranostic-based nanocarriers are illustrated in Figure 2. Nanotechnology offers significant potential for addressing existing issues and improving OVCA diagnosis and therapy. Although the nanomedical field is still in its early stages, we have recently seen an increase in research in this area, particularly with regard to cancer applications. Even though, the literature and current reviews on nanotechnology are primarily focused on nanomaterials or nanosystems, with an emphasis on their properties and broad applications in cancer and other diseases. The focus of this review is on the diagnosis and therapy of ovarian cancer using nanotehnological approaches. This review highlighted the potential bio-applications of various nanotechnology-based materials and their role in the treatment and diagnosis of ovarian cancer

Liposomes exhibit a lipid bilayer that holding hydrophobic interface that enables the attachment of therapeutic and diagnostic agents of both hydrophilic and hydrophobic nature to the geometry of liposomes. The core region of dendrimers is closer to the hydrophobic component and hydrophilic termini that helps in the delivery of hydrophobic payloads within water insoluble domain. On another hand chemical conjugation of moieties with the exterior is take place for its delivery. Adsorption of carbon nanotubes to various agents is take place for consecutive delivery. Micelle provides delivery platform for hydrophobic anticancer drugs with prolong circulation.

## 2. Ovarian cancer diagnosis via biosensors

In recent years, ovarian cancer is globally classified as deadly gynecological cancer (Boitano et al., 2019). It is believed that considerable public health and clinical consequences have been observed in the prevention and key variable detection of ovarian cancer that is attributed to weak diagnostic tests (Giamougiannis et al., 2021). Ovarian cancer is classified into stages I, II, III, and IV that play a key and crucial role during treatment (Macciò et al., 2020). At the initial stages, ovarian cancer poses certain specific symptoms and its early diagnosis is in favor of patient survival otherwise a small survival rate i.e. 8–30% occurs in ovarian cancer patients with advanced stages (Koo et al., 2020). Of note, the use of oral contraceptive drugs and obesity in populations have raised the incidence of ovarian cancer with a predicted mortality rate globally (Reid et al., 2017; Momenimovahed et al., 2019). As a result, the early stage detection of ovarian cancer is too much important. In the context of ovarian cancer biomarker identification, traditional diagnostic methods were applied that include polymerase chain reaction, radioimmunoassay, immuno-sorbent assay linked to enzymes and mass spectrometry (Anzar et al., 2020; Qian et al., 2020). However, these methods need more skilled staff, time-consuming data collection, sophisticated equipment, complicated/special pre-treatment of samples, costly, careful isolation and cleaning procedures are the main drawbacks associated with these conventional methods (Razmi & Hasanzadeh, 2018). To cope with these issues, a miniaturized and innovative sensing method is required that easily and quickly asses the ovarian cancer patients (Gasparotto et al., 2017; Pulikkathodi et al., 2018). Sensors are analytical instruments comprised of receptors, transducers, and reading systems and used to detect and measure the concentration of analytes in a sample. Specific interactions between the biological receptor and the analyte of interest are translated into a measurable signal by the transducer (Er et al., 2021) Medicines and biology-based nanotechnology are referred to as nano-biotechnology that evolves nanomedicines that are used for medical applications and this approach extends toward nanoelectronic sensors as well (Masson, 2017; Lee et al., 2020). Multiple nanoparticles with various features such as chemical characteristics and crystallinity configuration had resulted in its potential use in drug delivery (Sha & Badhulika, 2020). The development of specific drug carrier system is considered a significant platform for cancer therapy (Sivasankarapillai et al., 2021). In addition, the nanosensors convert information in analyte presence into signals that could be used for diagnostic purposes (Munawar et al., 2019).

Biomarkers manifest versatile class of biomolecules that indicates a specific disorder or biological function (Ueland, 2017). In the clinical diagnosis, the biomarker amount is trickled which helps in the diagnosis of various abnormalities in biological media (Ohmichi et al., 2018; Mithraprabhu et al., 2021). In the context of ovarian cancer, the significant biomarkers that indicate cancer includes, mucin-1, prostatin, cancer antigen CA-125, apoptosis repeat baculoviral inhibitor-5, human epididymis protein-4, and e-cadherin (Gray et al., 2016; Yang et al., 2020). In order, to distinguish various ovarian cancer biomarkers and provide a background for diagnosis few sensors are discussed here.

### 2.1. Optical nanosensors

**Figure 3.**
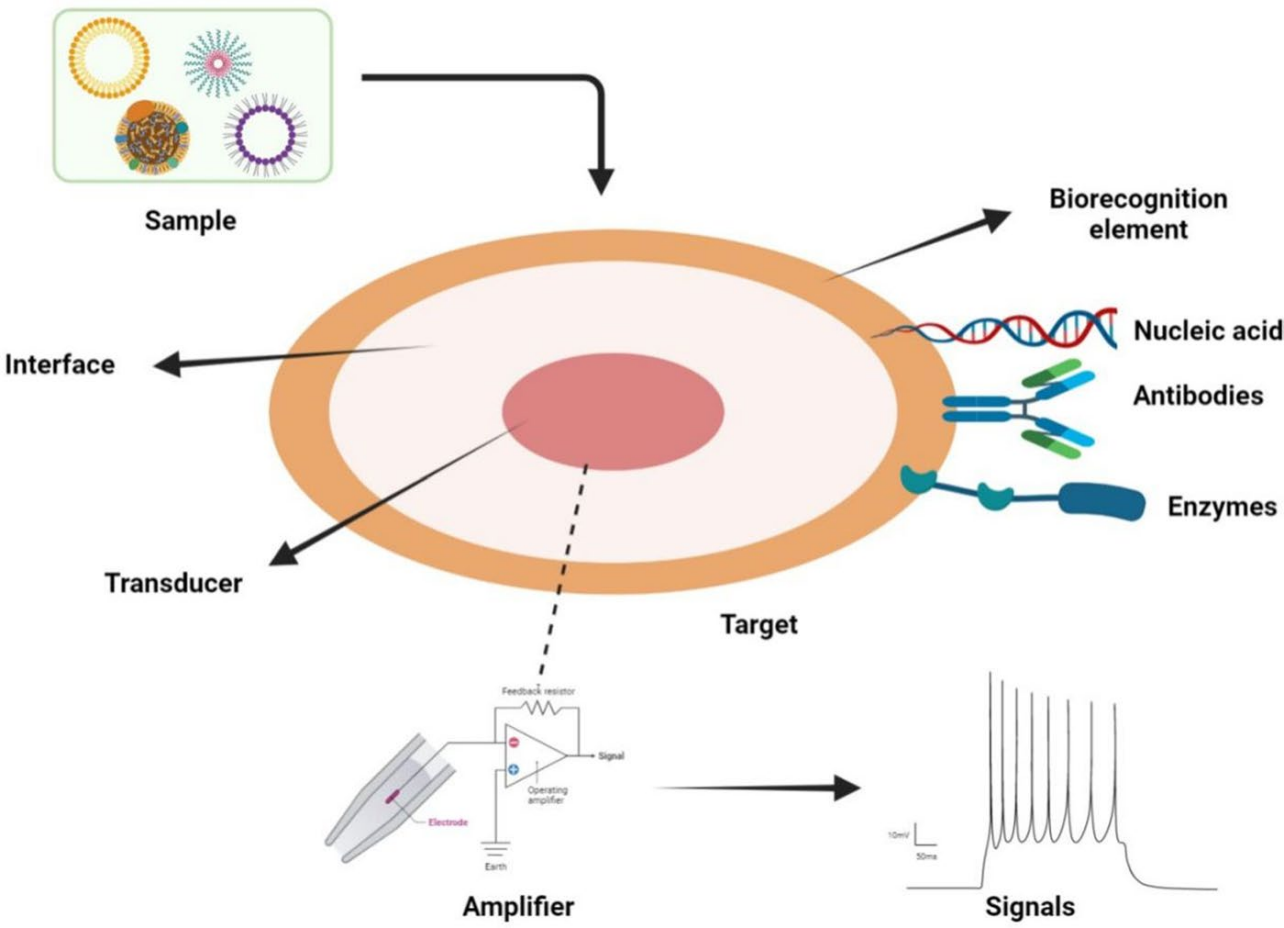
Schematic illustration of biosensor with optical biosensing applications.

Optical biosensor utilizes an optical transducer system with sensing features that are used for analysis purposes. It senses and transmits those signals that are directly proportional to the biomarker (Figure 3, Liu et al., 2016; Sohrabi et al., 2020). These biosensors are based on optical biosensors, surface Plasmon resonance and fluorescence (Soler & Lechuga, 2019).

**Figure 4.**
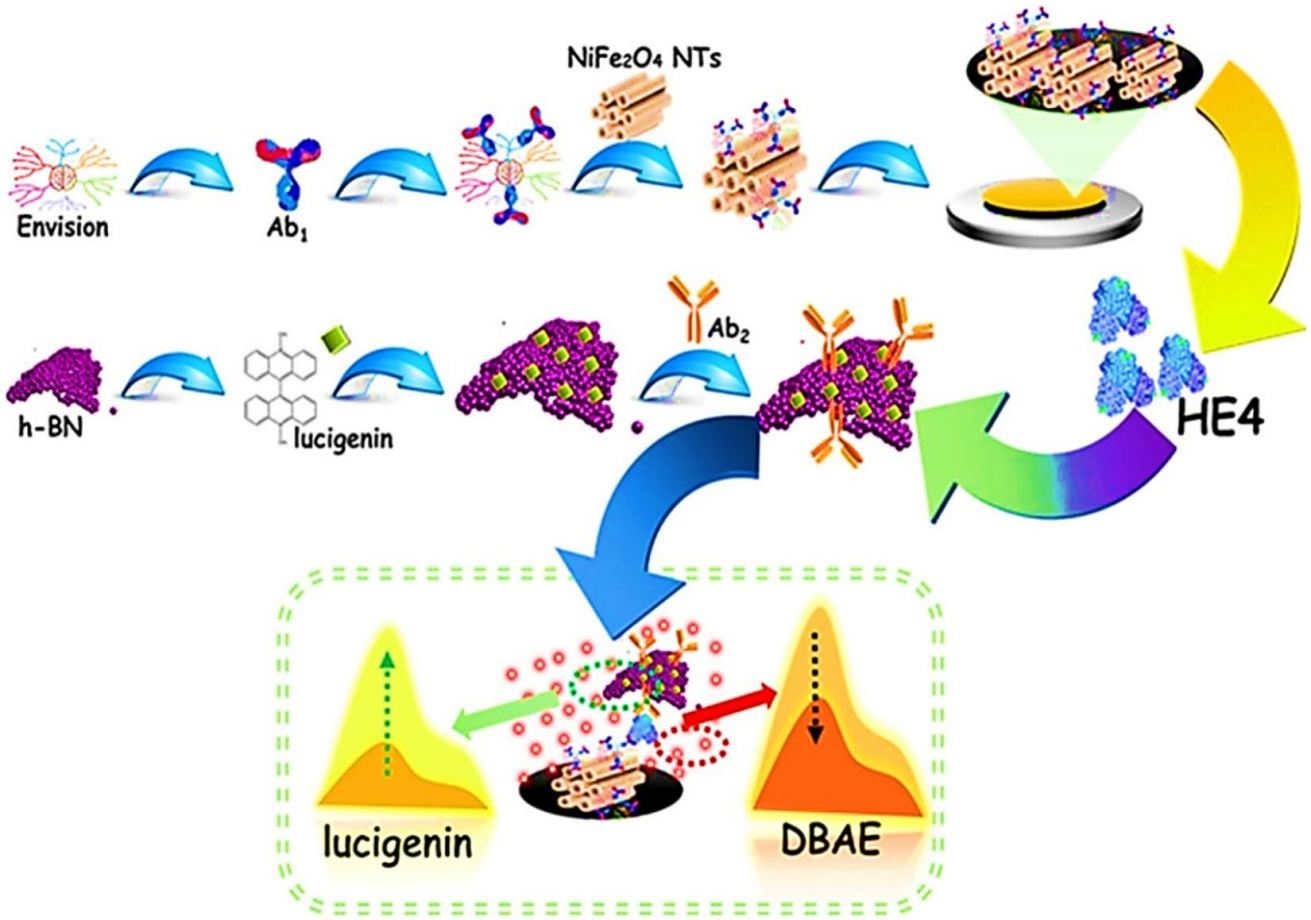
Schematic architecture of the NiFe_2_O_4_ nanotubes electrochemiluminescence nanosensor for HE4 detection as an ovarian cancer biomarker. Reproduced with permission from Wang et al. (2019).

To improve the surface glycosylation visibility of sample cells an accelerating technique was used that showed efficient results. During the technique the MUC1 precise glycosylation on the cancer cell surface was pictured through a quantum dots label using the strategy of RMC magnification. This study offers a sort of suggested scheme for protein-specific glycosylation and thus indicates the mentioned technique as a feasible strategy (Yang et al., 2019). Apoptosis repeat baculoviral inhibitor containing-5 was considered a significant biomarker for the ovarian cancer diagnosis and a negative growth correlation was observed with its overexpression (Smith et al., 2019; Wang et al., 2020). Furthermore, in a dog serum Apoptosis repeat baculoviral inhibitor containing-5, protein biomarker was detected through surface Plasmon resonance immuno sensor. The identified biomarker quantity was found less such as 6.2 pg/mL, along with 73% sensitivity and, 95% specificity. These findings from dog sera pave ways for onsite nano sensing in patient testing (Jena et al., 2019). In another study, an immunoassay was developed for the identification of CA-125, biomarker in ovarian cancer. The detection limit observed was in a linear range of 0.10–600 U/mL for CA-125 (Al-Ogaidi et al., 2014). Similarly, anomalous epididymis protein-4, biomarker was identified through ratiometric electrochemiluminescence nanosensor in ovarian cancer. Results showed biomarker detection in a limit of 3 fg/mL and estimation range between 10 fg/mL and 10 ng/mL (Figure 4, Wang et al., 2019).

### 2.2. Electrochemical biosensors

**Figure 5.**
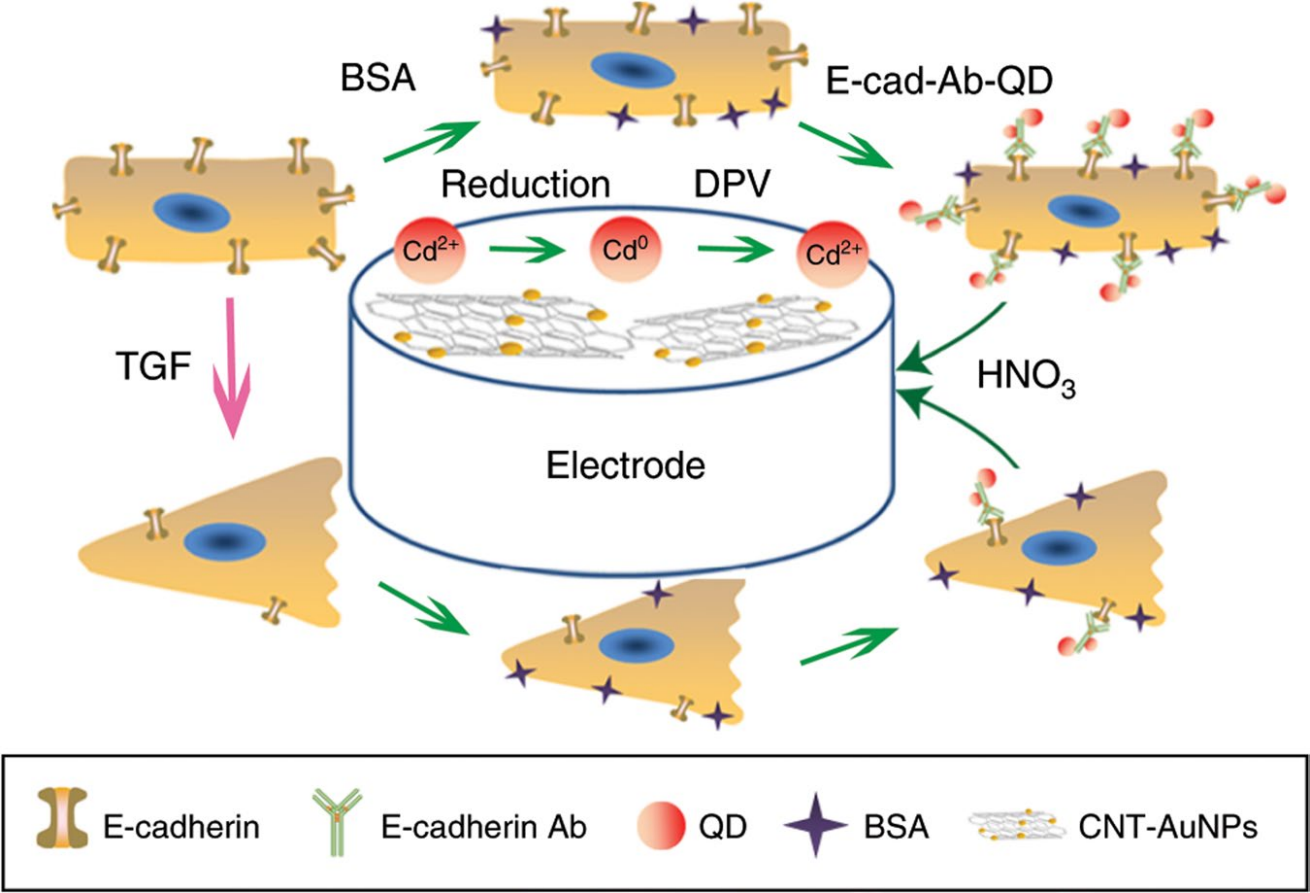
Design of the electrochemical nanosensor for low detection of E-cadherin as an ovarian cancer biomarker (Du et al., 2020).

Electrochemical nanosensors have shown potential methodology and role for the precise detection of biomarkers in ovarian cancer and usually have the detection power in minute quantities of biomarkers and other analytes (Parmin et al., 2021). In the identification and detection of ovarian cancer biomarkers electrochemical sensors were used in combination with nanoparticles that provided multipathing abilities along with enhanced sensitivity (Barani et al., 2021). In 75% of patients, the existing diagnostic strategies have resulted in the identification of CA-125, biomarkers in stages I and II ovarian cancer. Such early stage detection and diagnosis could be boosted up via low concentration tracking of CA-125 biomarkers (Charkhchi et al., 2020). Similarly, graphene nanosensors were developed by a research group for the detection of CA-125 biomarkers in order to ensure label-free detection following polyaniline surface precipitation and conjugation with anti-CA 125 antibodies. In that particular development era the developed nanosensor was most sensitive detective device for CA-125 with a detection limit of 0.92 ng/µL (Gazze et al., 2018). Among other biomarkers, e-cadherin was also used as a tumor detective biomarker because its expression was found in negative association with the presentation and recognition of ovarian cancer (Rea et al., 2018). Du et al. conducted a study on detection of ovarian cancer biomarkers by using quantum dot and carbon nanotubes nanocomposites as electrochemical nanosensors based on e-cadherin changes detection. Results showed responsive and rapid electrochemical signal transduction attributed to the synergistic effect of applied nanomaterials Design of the electrochemical nanosensor for low detection of E-cadherin as an ovarian cancer biomarker (Figure 5, Du et al., 2020).

**Figure 6.**
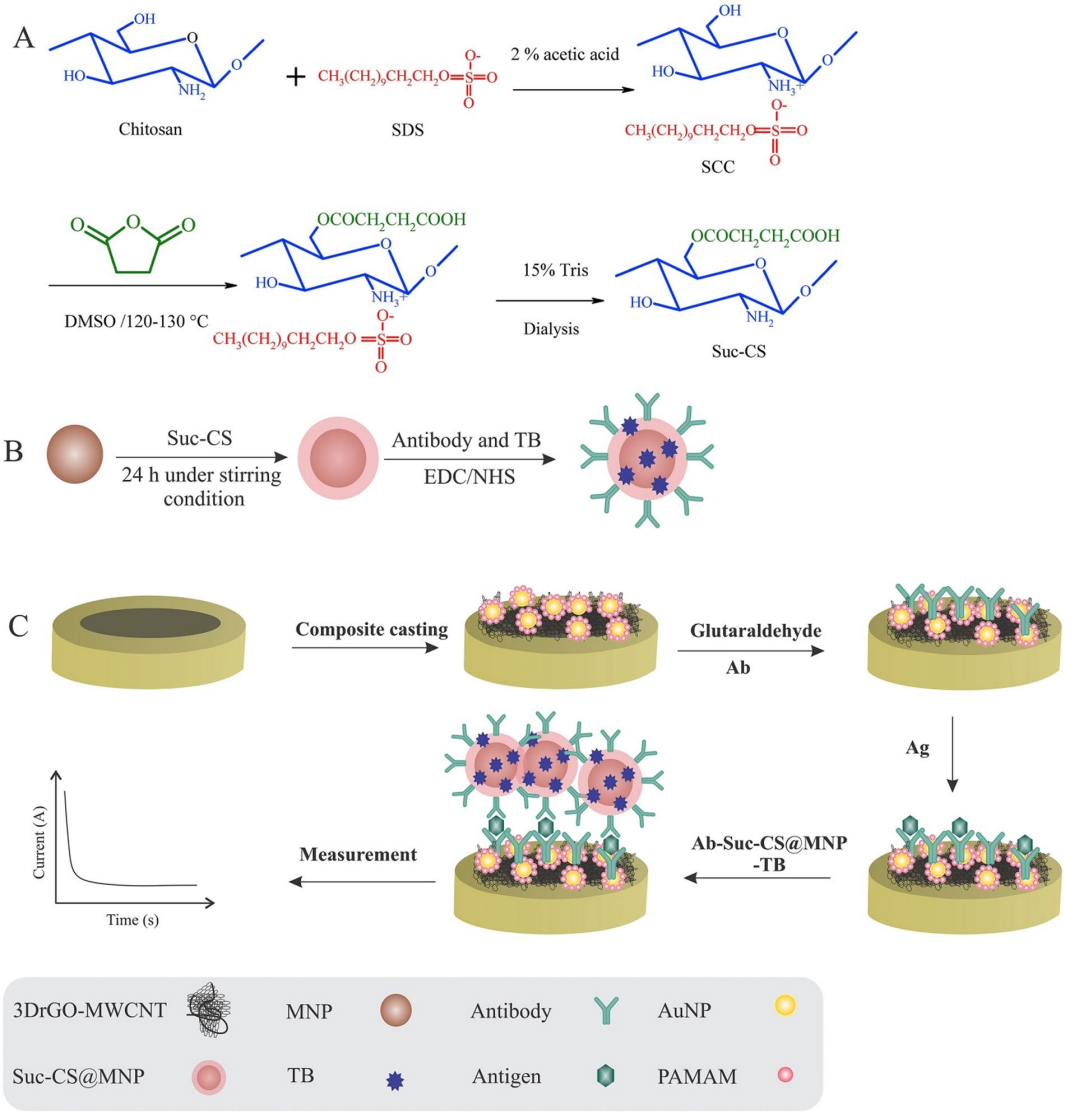
Construct of nanosensor based on polyamidoamine/gold nanoparticles (PAMAM/AuNPs) and 3D reduced graphene oxide-multiwall carbon nanotubes (3DrGO-MWCNTs) for the detection of biomarker CA-125. Reproduced with permission from Pakchin et al. (2020).

Another research group worked on the CA-125, biomarker identification through the development of immuno-based electrochemical nanosensor. The developed nanosensor was composed of multiwall carbon nanotubes, gold nanoparticles and 3D reduced graphene oxide composite. The developed system displayed an excellent detection limit i.e. 6 µU/mL (Figure 6, Pakchin et al., 2020).

### 2.3. Nanofluidics nano biosensors

Micro fluidic laboratory-on-chip nanosensor integrates easily the various features of multiple sensing systems with less sampling rate and thus pose it a versatile miniaturized system (Jamshaid et al., 2016). It produces cost-effective devices because of utilizing minimum resources and reagents and offers an efficient and high rate of biomolecules identification (Akceoglu et al., 2021). Within body fluids the existence of exosomes ensures the signaling of various cells and such nanoscale particles are manifested by biomolecules as well (Boriachek et al., 2018). In this context, based on biomarkers it has been reported that microfluidic system has the capability of capturing exosomes efficiently, however, for the testing purpose the release of captured exosomes is quite challenging (Wu et al., 2017). An antibody functionalized microfluidic platform was reported for the detection of biomarkers that exist in the membranes of cancer exosomes, i.e. EpCAM, CD9, in order to dissociate exosomes from ovarian cancer serum. The intact exosomes were significantly released and ultimately showed downstream internalization toward ovarian cancer cells (Hisey et al., 2018). In order to extract exosomes from clinical specimens and culture media a novel microfluidic system was developed in which sample isolation was centered on epithelial cell adhesion molecule and high precision CD63 expression that additionally resulted to avoid the problem of contamination. The designed microfluidic system helped in the diagnosis of serous ovarian cancer of high-grade nature particularly in women with HGSOC (Dorayappan et al., 2019). It is reported that the conventional in vitro experiments are unable or it’s a quiet hectic job to identify phenotypic heterogeneity and spatial metabolic of the tumor environment. In this context, the cell activity was studied under metabolic starvation using a slice model of microfluidic tumor and the findings of the study suggested that the designed system was composed of a central core containing 3D collagen hydrogel in which the tumor cell was grown. To test molecular adaptations the microdevice was capable of recovering the cells after its disassembly. The up-regulation of certain genes associated with DNA repair and replication displayed a response toward survival. It was concluded that the developed system would help in the targeting of metabolic heterogeneity and opening horizons in therapeutic possibilities in the arena of solid tumors (Ayuso et al., 2019).

### 2.4. Magneto resistive and paper based biosensors

Within a portable system, it is the unique feature of giant magnetoresistive biosensors that it measures multiple biomarkers all together. In addition, other advantages include integrated circuit connectivity, high accuracy and easy biomolecular detection (Xuan et al., 2021). In a research study, a portable giant magneto resistive nanosensor was developed aimed for the diagnosis of different cancers along with its concerned protein biomarkers. The developed system significantly showed multi-detection of CA-125 II cancer antigen (3.6 U/mL), interleukin (7.2 U/mL), and epididymis protein 4 (7.3 U/mL, Klein et al., 2019). Another type of nanosensors used for the diagnosis of ovarian cancer is paper-based biosensors that have attained greater attention due to its bioactivity, cheapness, availability and reusability. Photolithography, wax and screen printing are various techniques used for the development of paper-based biosensors (Fan et al., 2019; de Castro et al., 2020). To detect CA-125 biomarker in ovarian cancer paper-based nanosensors was prepared by absorbing anti-CA-125 antibody on the nano matrix surface. Graphene-based quantum dots immobilized with silver nanoparticles were used in matrix formation. Findings showed that under ideal conditions the low detection limit was 0.001 U/mL with linear range of 0.001–400 U/mL (Saadati et al., 2020). To diagnose initial stage ovarian cancer based on CA-125 detection paper based nanosensor was used following electrochemical techniques caped with gold nanoparticles. The suggested nanosensor showed a low detection limit of 0.78 U/mL with 0.7–400 U/mL linear range (Bahavarnia et al., 2019).

## 3. Nanomaterials in ovarian cancer treatment

In the recent years, many nanoconjugates and nanoformulations as a drug delivery system have been developed and they have improved the therapeutic drug delivery to the target side due to unique features such as biocompatibility, high therapeutic efficacy as compared to free drug, non-toxicity, biodegradability, off-target side effects and ease of manufacturing scale-up (Larrañeta et al., 2018). The prepared nanosystem in chemotherapy should have high drug dissolution, efficient drug loading capacity and desired target site accumulation through the influence of enhanced permeability and retention (Pantshwa et al., 2020; Zhao et al., 2020). Therefore, multi-functionalized nanomaterials have revolutionized the era of cancer diagnosis and treatment for targeted action via the attachment of biocompatible and specified ligands to target the tissues that are over-expressed in certain cancer types (Arshad et al., 2021).

### 3.1. Liposomes

Liposomes are nontoxic phospholipids-based sphere-shaped small vesicle ranges in size from 400 nm to 2.5 mm. Liposomes are extensively employed renowned clinically delivered nanosystems with biodegradable nature and have the capability to entrap hydrophobic and hydrophilic biomacromolecules, i.e. RNAs, peptides and proteins without modification in their inherent properties (Sun et al., 2017; Janani et al., 2022). To avoid the liposomal elimination by phagocytic system and improve their circulation, PEGylation is carried out. In this regard, paclitaxel-loaded PEGylated liposome nanoformulations were fabricated and evaluated in the in vitro and in vivo model of ovarian cancer cells in order to suppress the cancer cell multiplication. The ovarian cancer cell aggressiveness was inhibited markedly after treatment with a fabricated nanosystem. Moreover, in ovarian cancer cells the caspase 3/9, ERK was highly expressed that resulted in apoptosis induction (Qi et al., 2018). Similarly, in another study PEGylated liposomes were developed for the loading of cisplatin with an aim to explore the cellular uptake of cisplatin in sensitive as well as resistant ovarian cancer cells through transferrin receptor targeting. In resistant cells, after 24 hours the free drug uptake was four-fold reduced and the transferrin receptor targeting was insignificant that strappingly necessitates drug delivery based on liposomes in cisplatin-resistant evasion (Krieger et al., 2010).

The co-loading of two chemotherapy agents into a single liposome in clinical trials has shown many advantages but the loading of two chemotherapeutic drugs into a single liposome is quite challenging. In this context, irinotecan and doxorubicin were encapsulated and loaded into liposomal nanoformulations to treat ovarian cancer xenograft. Various ratios between drugs to the drug were used during loading into the liposome. The encapsulation efficiency was 80%, and it was found that after storage for 6 months the liposomal stability attributes significantly influenced the drug encapsulation. After the administration of developed nanosystem via intraperitoneal route considerably enhanced the survival of the animals with tumor, which was attributed to increased exposure of liposomal nanoformulations to the systemic circulation (Shaikh et al., 2013). In another study, folate-capped liposomes were designed and targeted to intraperitoneal ovarian cancer cells exhibiting folate receptors. Results showed that the applied nanoformulation was taken up by tumor-associated macrophages assisted by folate receptor internalization. The engulfment was 10-fold high by macrophages as compared to tumor cell ascites (Turk et al., 2004).

### 3.2. Nanoparticles

Several nano-sized drug delivery systems have been tested in cancer treatment to reduce the side effects of traditional anticancer drugs while increasing the antitumor efficacy of target therapy (Mohammadzadeh et al., 2022). In ovarian cancer therapy, the fabrication and development of metal nanoparticles have received greater attention. The modification and synthesis of such nanoparticles have made it an attractive nano-based approach that is based on size, shape and target site accumulation (Barani et al., 2021). In addition, among metal nanoparticles, iron-oxide-based nanoparticles have shown anticancer potential (Arakha et al., 2015; Khatami et al., 2019). In this connection, a wet chemical procedure was used for the fabrication of irregular shape and monocrystalline iron nanoparticles and was evaluated in metastatic human ovarian cancer cell line (PA-1 cell). Invitro cytotoxic results demonstrated an enhanced intracellular reactive oxygen species level, apoptosis and mitochondrial membrane destabilization (Ramalingam et al., 2020). It is believed that the superparamagnetic features of metal nanoparticles impart exceptional properties to metal nanoparticles and thus could be used in nanostructures extensively for ovarian cancer (Chan et al., 2019).

**Figure 7.**
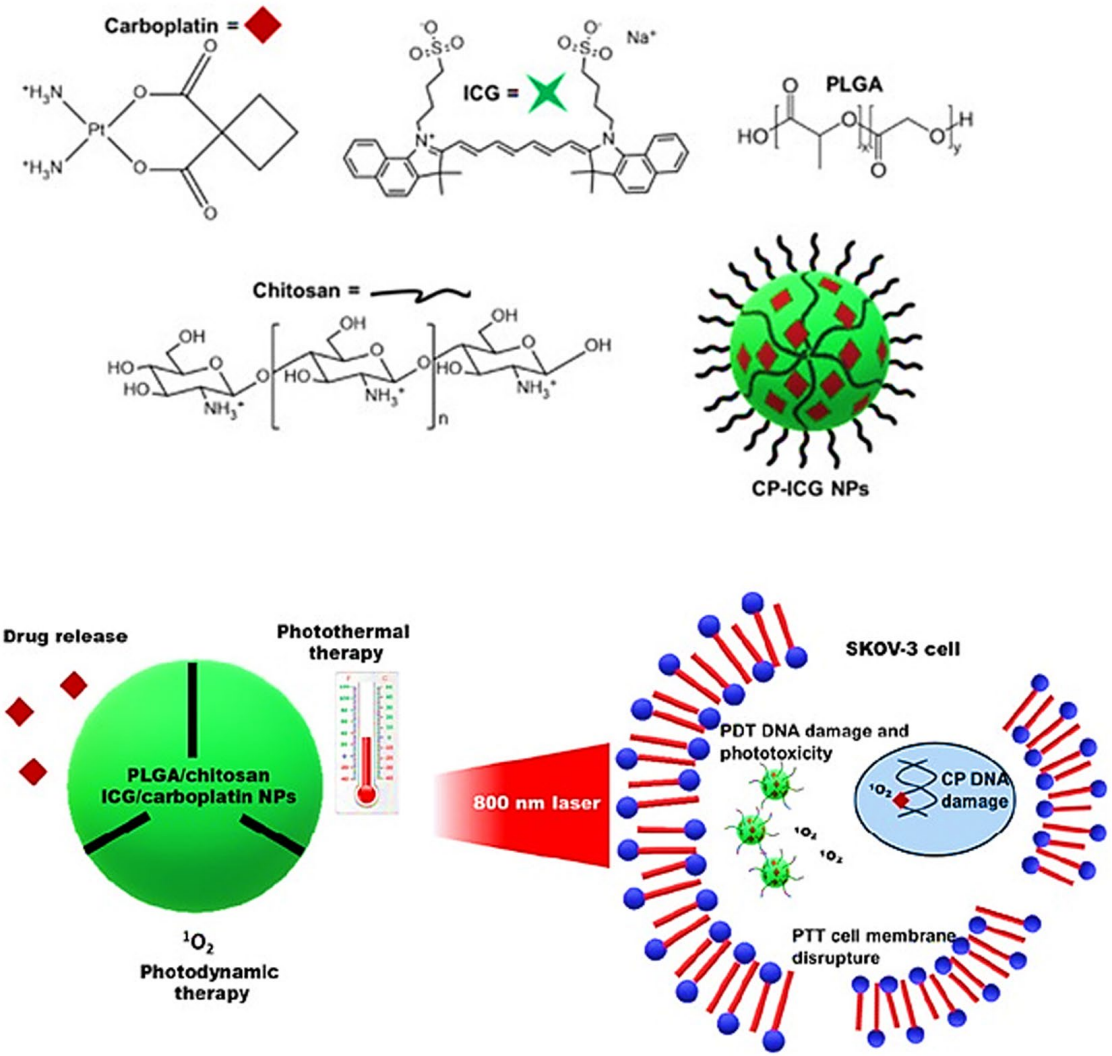
Schematic illustration of CP-ICG NPs combining chemo-, photothermal-, and photodynamic therapeutic abilities. Reproduced with permission from Sánchez-Ramírez et al. (2020).

In addition to metal nanoparticles, biomacromolecules such as vitamins, nutrients, hydrophobic drugs and phenolic compounds were immensely delivered to multifaceted biological systems using chitosan nanoparticles. Chitosan-a chitin-deacylated biopolymer of natural origin that exhibit useful features such as inertness, biodegradability, biocompatibility and the presence of hydroxyl and amino group that make it a suitable candidate for the drug delivery (Muddineti et al., 2017). The affinity of hydroxyl groups for hydrogen bonding and the creation of linkages by amines with acidic drugs have made chitosan a versatile polymer that during functionalization has opened avenues in the novel drug delivery system. In this context, surface functionalization with chitosan and the consecutive attachment of nucleic acid, protein and drugs with its amino group resulted in size adjustment in a range of 200 nm along with an efficient accumulation of therapeutic agents at tumor microenvironment and augmented EPR effect (Miao et al., 2018). In this context, a chemotherapeutic and photoactive drug-loaded chitosan-based biodegradable and biocompatible nanosystem was developed and evaluated in SKOV-3 ovarian cancer cell lines. Results showed an enhanced antitumor and cytotoxicity efficacy against these cell lines. In association, biodegradability, biocompatibility, spatiotemporal control of therapy and lack of resistance associated with chitosan material supported the application of such fabricated nanoparticles in clinical trials (Figure 7, Sánchez-Ramírez et al., 2020).

In another research study, cannabidiol-loaded polymeric nanoparticles were developed and evaluated in ovarian cancer cell for growth inhibition potential. The encapsulation efficiency found was 95% with 240 nm size nanoparticles and displayed controlled release kinetics for cannabidiol after internalization to SKOV-3 ovarian cancer cells for an extended period of 96 hours. After encapsulation, the antiproliferative effect of cannabidiol was found excellent against the SKOV-3 cells. Apoptosis was also induced by the applied drug-loaded nanosystem that was confirmed when PARP protein was found provoked. Importantly, the ovarian tumor growth inhibition was slightly higher for cannabidiol-loaded nanoparticle as compared to free cannabidiol (Fraguas-Sánchez et al., 2020). These findings suggest that chitosan and PLGA polymeric-based nanoparticles could provide a novel and efficient approach for the delivery of therapeutic candidate intraperitoneally targeting ovarian cancer.

In the arena of ovarian cancer targeted drug delivery epigallocatechin gallate-loaded chitosan base mesoporous silica nanoparticles were developed and evaluated. Furthermore, the fabricated system was attached with AS1411 aptamer through conjugation with the amine group present in the chitosan using electrostatic attraction. The aptamer functionalized nanosystem followed macro-pinocytosis and displayed efficient internalization potential in SKOV-3 cell lines. Consequently, it resulted in an augmented cytotoxic effect in 93% of ovarian cancer cells along with G1 cell cycle arrest and down-regulation of ERK2 expression level (Alizadeh et al., 2020). It is concluded that aptamer-mediated nanoparticle delivery from nanotechnology platform has proven its potential in ovarian cancer task-specific targeting. Similarly, thymoquinone-loaded radio iodinated folic acid nanostructures based on chitosan were fabricated and evaluated in SKOV3 ovarian cancer cell lines to improve and demonstrate its targeting efficiency. Results showed that as compared to free thymoquinone the chitosan-based nanostructure counterpart showed higher incorporation efficiency in ovarian cancer cells. In association, prominent changes in SKOV3 cells plasma membrane were observed that provide evidence for the target-specific targeting of ovarian cancer cells by fabricated nanoparticles (İnce et al., 2020).

### 3.3. Solid-lipid nanoparticles

Solid-lipid nanoparticles are made up of surfactants, lipids and therapeutic drugs enclosed in spherical shape colloidal nanocarriers exhibiting 50–1000 nm diameters. The lipid core and nano size range of solid-lipid nanoparticles make them versatile drug delivery carriers from nanotechnology platforms. Such nanocarriers have attained greater interest also due to their considerable biocompatibility, excellent circulation time and marked accumulation at the target site (Radhakrishnan et al., 2016; Naidoo et al., 2018). In the context of cancer treatment, paclitaxel-loaded solid-lipid nanoparticles were developed for the treatment of the human ovarian cancer cell line (OVCAR3). Results of the comparative analysis from commercial preparation showed a similar cytotoxic response after intravenous administration concluding lipid-solid nanoparticle as a suitable novel drug delivery system (Lee et al., 2007). Verteporfin is a clinically registered photosensitizer drug, its solid-lipid-based nanoparticles were fabricated for ovarian cancer treatment. After laser irradiation, the developed nanosystem was efficiently internalized into the tumor environment and as a result as strong suppression of tumor cell viability was achieved. The longer circulation of verteporfin was also observed along with effective tumor uptake that was further confirmed from biodistribution and pharmacokinetic profile. Of note, adverse effects along with toxicity associated death were observed with free drug administration at a dose of 2 mg/kg; however, drug-loaded solid-lipid nanoparticles resolved that issue and after intravenous injection of nanoformulation showed no toxicity at a dose of 8 mg/kg. In addition, tumor proliferation was inhibited significantly at such dose of drug-loaded to solid-lipid nanoparticles (Michy et al., 2019). Recently, ovarian cancer was targeted via intraperitoneal therapy through paclitaxel-loaded solid-lipid nanoparticles. As compared to free drug the drug-loaded nanoformulation showed enhanced cytotoxicity. The fabricated solid-lipid nanoparticles achieved a long circulation time in Wistar rats attributed to the slow absorption of drug-loaded nanoformulation (Han et al., 2019). All these findings suggest the applicability of solid-lipid based nanoparticles in ovarian cancers as well as other peritoneal cancers.

### 3.4. Dendrimers

Dendrimers are spherical nano size, regularly branched, three-dimensional tree-like architectures of high molecular weight in which the branch lengths are stearic limited exhibiting an inner core. The inner microenvironment and surface of dendrimers are composed of functional moieties that help in the attachment of delivering cargoes (Mishra et al., 2019; Mittal et al., 2021). Dendrimers usually hosts both hydrophobic and hydrophilic moieties and thus considered prodigious nanoplatform for drug delivery. Over the past few years, many researchers attempted efforts in the field of dendrimers in order to overcome the issue of cytotoxicity and promote its rapid clinical translation (Janaszewska et al., 2019). In this regard, cisplatin-loaded dendrimers were evaluated for its anticancer potential that resulted in no cytotoxicity. In addition, relative to the control group tumor size was reduced by 33% after administration of 6 mg/kg dose of free cisplatin however; 45% reduction in size was observed for cisplatin-loaded dendrimers after administration at a dose of 6–8 mg/kg (Kirkpatrick et al., 2011). In the context of ovarian cancer, both hydrophobic and hydrophilic chemotherapeutic drugs (paclitaxel and cisplatin) loaded telodendrimer were developed and evaluated for synergistic effects in SKOV-3 ovarian cancer cell line. On one side, as compared to free drug the loading of single drug-based telodendrimer showed less cytotoxic effect that was attributed to the slow drug release profile. On another hand, the co-loading of cisplatin and paclitaxel in an optimal ratio of 2:1 into telodendrimer displayed an enhanced cytotoxic effect that was attributed to their synergistic effect. Results from imaging analysis suggested 4 fold more accumulation of drugs at the target site i.e. SKOV-3 ovarian cancer xenograft relative to other organs (Cai et al., 2015). Recently, non-hemolytic and non-toxic folate targeted polyurea dendrimers were developed that aimed the stemming of chemoresistance in ovarian cancer considering it as a new weapon in ovarian carcinoma. The suppression of glutathione synthesis on repair and renewal of ovarian carcinoma sensitivity to carboplatin was evaluated and the effects were observed via in vitro study. After treating cells with l-buthionine sulfoximine—a γ-glutamylcysteine ligase inhibitor, the glutathione synthesis was hampered significantly. Systemic toxicity consideration in this context helped in overcoming the issue of carboplatin resistance by using polyurea-based dendrimer formulations (Cruz et al., 2020). These findings suggest that dendrimers based nanoformulation hopefully would reinstate the responsiveness of ovarian cancer cells toward chemotherapeutics.

### 3.5. Nanomicelles

Among nanostructured materials micelles represent an intriguing class of nanoarchitecture in which amphiphilic molecules above critical micelle concentration aggregates to form such a versatile class of nanocarriers (Karayianni & Pispas, 2016). Structurally, it is composed of an amphiphilic block co-polymer and core–shell that hosts various hydrophobic and hydrophilic therapeutic drugs and carries efficiently toward the target site (Zhang et al., 2017). In the treatment of ovarian cancer, micelle are considered potential nanocarriers due to remarkable chemotherapeutic drugs loading capacity and then efficient target-specific targeting capability (Yu et al., 2018). It was reported that a 10–100 nm range micelles reduce normal cell non-specific targeting due to enhanced penetration power and ultimately endocytosis toward ovarian cancer cells (Li et al., 2017). Some unique features of nanomicelles such as tumor perforation, high biocompatibility, hydrophobic chemotherapeutic loading, in vivo stability and extended circulation in plasma make them prosing nanocarriers in the treatment of ovarian cancer (Shariatinia, 2021). Paclitaxel-loaded redox-sensitive nanomicelles were developed for the treatment of ovarian cancer that was chemo resistant. Following a redox-sensitive manner, the ovarian cancer cells SKOV-3 were treated through such micellar nanosystem (Mutlu-Agardan et al., 2020). Similarly, to evaluate the pharmacokinetics and cytotoxicity of docetaxel, its folate targeted nanomicelles were fabricated and evaluated in SKOV3 ovarian cancer cell lines. Results showed that as compared to free drugs the docetaxel-loaded micellar system displayed high cytotoxicity (Kazemi et al., 2021). Recently, polymeric micelle fabrication was reported that was aimed for determining of high loading capacity of two hydrophobic drugs i.e. irinotecan and doxorubicin. Between the drugs and polymeric micelles a tunable ratio was adjusted and a donor-receptor interaction was considered. The drugs loaded micelles showed drug high ultra loading, desired size distribution, efficient biocompatibility, significant ovarian cancer cell uptake, profound stability and more importantly the reactive oxygen species overproduction that resulted in the effective release of loaded cargoes in the cancer cell environment. In addition, the anti-ovarian cancer activity was also provoked significantly by such developed micellar system confirmed from both in vitro and in vivo studies (Wu et al., 2020). This finding reveals that the use of controllable drug ratio precisely and incorporation of drugs with high ultra loading into the micelles-based nanocarriers ensures an excellent synergistic activity from chemotherapeutic agents in ovarian cancer therapy. In association, such nanoformulations have displayed negligible cytotoxicity and thus could be used as suitable delivery platforms for many other therapeutic candidates i.e. antibiotics, proteins, etc.

### 3.6. Nanocapsules

Nanocapsules manifest a nanoscale vesicular system that is composed of central cavity that gives space to the drugs of interest. In addition, an outer polymeric shell is also there surrounding inner core and it helps in the attachment of various targeting ligands and moieties during surface functionalization (Li et al., 2022). Due to its protective coating property such as in drug delayed release, pyrophoric and readily oxidized has made it subject of high interest. To obtain nanocapsules various methods were applied however; nano-deposition and interfacial polymerization are preferably utilized approaches for the fabrication of nanocapsules. Nanocapsules were used for controlled release of drugs targeting ovarian cancer and that is attributed to its high and extreme reproducibility. In addition, the target-specific and enhanced active drug delivery through nanocapsules opens new array of novel opportunities in order to design advanced drug delivery systems for the ovarian cancer treatment (Haggag et al., 2020; Wang et al., 2020). In this connection, in mice carrying OVCAR 3 cells—human ovarian carcinoma cell line, cisplatin-loaded nanocapsules conjugated with PEG were evaluated for its anticancer potential. Results showed that after 20 days of initial injection the fabricated nanosystem displayed 90% growth reduction in ovarian tumor. Albeit, the nanoformulations in comparison to free cisplatin showed growth inhibition in xenograft mice (Staffhorst et al., 2008). To tackle the issue of multi drug resistance, paclitaxel–lapatinib loaded nanocapsules in the treatment of ovarian cancer showed and enhanced growth inhibitory activity attributed to the efficient delivery of cargoes into the target site (Barani et al., 2021).

A summary of various nanosystems is provided in Table 1 which are based on different characterization parameters and nature of application.

**Table 1.** List of various nanocarriers used in ovarian cancer treatment and diagnosis.

Nanoparticles	Drug/antibody	Characterization			Application	References
		Size	PDI	Zeta potential		
Polyamidoamine/gold nanoparticles	Anti-CA125 Ab	14 nm	N/A	N/A	Electrochemical immunosensor for ultrasensitive detection of CA125 in ovarian cancer	Pakchin et al. (2020)
Paper-based immune device modified with cysteamine caped Au nanoparticles	Anti-CA 125 antibody	20 nm	N/A	−29.1 mV	Paper based immunosensor for efficient diagnosis of ovarian cancer	Bahavarnia et al. (2019)
Peptide-modified lipids liposomes	Paclitaxel	109.97–134.90 nm	0.240–0.268	−6.14 ± 0.62 and −11.11 ± 1.65 mV	Cancer treatment	Sun et al. (2017)
PEGylated liposomes	Cisplatin	110 nm	N/A	N/A	PEGylated liposomes for overcoming cisplatin resistance of ovarian cancer	Krieger et al. (2010)
Liposomes	Doxorubicin and irinotecan	111 nm	0.15	N/A	Synergistic combination of irinotecan and doxorubicin for the treatment of ovarian tumor	Shaikh et al. (2013)
Folate capped liposomes	N/A	65–90 nm	N/A	N/A	Targeting macrophages associated with ovarian carcinoma	Turk et al. (2004)
Hematite α-Fe_2_O_3_	N/A	28 nm	N/A	N/A	Treatment of human metastatic ovarian cancer	Ramalingam et al. (2020)
Chitosan/PLGA nanoparticles	Carboplatin	180.3 ± 1.1–222 ± 1.1 nm	N/A	−31.2 ± 2.1 to +31.8 ± 6.2 mV	Chemo-photodynamic therapy of ovarian cancer	Sánchez-Ramírez et al. (2020)
PLGA nanoparticles	Cannabidiol	240 nm	0.165 ± 0.009	−16.6 ± 1.2 mV	Treatment of ovarian cancer	Fraguas-Sánchez et al. (2020)
Chitosan-coated silica (SiO_2_@CS)-aptamer nanoparticles	Epigallocatechin gallate	257 nm	1	+7.14	Targeted delivery of EGCG to the SKOV-3	Alizadeh et al. (2020)
Folic acid-thymoquinone-chitosan nanoparticles FATQCSNPs	Thymoquinone	344.5 ± 28.64 nm	N/A	−9.05 ± 0.16 mV	Targeted delivery to ovarian cancer cells	İnce et al. (2020)
Solid lipid nanoparticles	Epigallocatechin gallate	157 nm	0.268 ± 0.14	−37.2 ± 2.5	Cytotoxicity against cancer cell lines	Radhakrishnan et al. (2016)
Paclitaxel-loaded sterically stabilized solid lipid nanoparticles	Paclitaxel	200 nm	N/A	−38 mV	*In vitro* cytotoxicity against human ovarian and breast cancer cell lines	Lee et al. (2007)
Nanostructured lipid carriers	Verteporfin	47.9 ± 1.0 nm	0.12 ± 0.02	−3.7 ± 0.9	Targeted photodynamic therapy of ovarian cancer	Michy et al. (2019)
Core-shell-structured solid lipid microparticles	Paclitaxel	1.76 ± 0.37 µm	0.21 ± 0.2	−19.54 ± 0.61 mV	Treatment of ovarian cancer	Han et al. (2019)
Poly(amidoamine) dendrimers	Cisplatin	2.7–5.9 nm	N/A	N/A	Cytotoxicity study against ovarian cancer cell lines	Kirkpatrick et al. (2011)
Three-layered linear-dendritic telodendrimer micelles	Cisplatin	16.9 ± 4.8	N/A	3.1	Synergistic combination nanotherapy for ovarian cancer treatment	Cai et al. (2015)
Pegylated multifunctional pH-responsive polymeric micelles	Docetaxel	88.8 nm	<0.3	−17.44 mV	pH-triggered folate targeted polymeric micelles for the treatment of ovarian cancer	Kazemi et al. (2021)
Polymeric micelles	Doxorubicin and irinotecan	30–40 nm	N/A	N/A	Co delivery of dual chemo-drugs for synergistic anti-cancer therapy	Wu et al. (2020)

## 4. Challenges and opportunities

The knowledge and research on nanotechnology-based formulations has bloomed in recent years, however, only few formulations have made their way successfully to clinics. Most of such formulations fail to demonstrate similar results when tested in vivo and halt their progress to clinical trials. For clinical translation, every nanoformulation has particular challenges, however, most of them confront biological, technological, and study-design-related challenges.

Biological challenges include lack of routes of administration, tempering biodistribution, hannelling nanosystems across biological barriers, toxicity and degradation (Lv et al., 2012). Often, intravenous injections of nanoformulations into the blood remove them from the target site. So, an excessive drug concentration is used, which may not have the desired effects (Ryman-Rasmussen et al., 2006). However, certain in vivo and in vitro studies have shown that magnetic nanoparticles can be used to control their movement against blood flow. However, more study into the impact of magnetic fields on humans, as well as the interaction between different magnetic fields and the presence of numerous nanoparticles, is required.

Controlling nanoparticle’s biological fate is difficult and requires focus. There is a possibility of liver, lung, and kidney damage, despite the fact that nanoparticles are made of biosafety materials. Surface area, shape, solubility, particle size and agglomeration are the factors that are reported to cause toxicity (Jia et al., 2018). Deposition of anosystems in lung exhibited inflammation, oxidative, and cytotoxic effects (Jiang et al., 2009). Healthy cells are often damaged by nanoparticle-generated free radicals (Awasthi et al., 2016). Possible solutions include making nanoparticles out of more biocompatible materials, like chitosan, or ones that break down when exposed to near infrared light.

Technological challenges of nanosystems include equal optimization, performance predictions and scale-up synthesis. Most nanoparticles used are produced in small batches, and scaling up is not always possible due to instrumentation and other factors. In animal models, the best lead clinical candidates are not always systematically designed and optimized. To circumvent this, we can employ specific methods that permit the testing of numerous nanoformulation and the selection of a single optimized formulation via selective iterations (Dobrovolskaia et al., 2008; Xia et al., 2020). Nanoparticle efficacy and performance are difficult to predict, and replicating in vivo results in human trials is difficult. Computational or theoretical modeling can imitate physiological tissue and surroundings. For example, organs-on-chips can strengthen NP predictions of efficiency and performance.

Study-design challenges such as study size, intent, and timing of nanoparticle therapies during therapy have a significant impact on clinical studies. The majority of research is based on “cell and animal models,” which may not translate to human trials. Therefore, it is challenging to mimic natural human body reactions using a single model. Metastasis is an important attribute of cancer, so “models of cancer metastasis” should be researched. *N* = 1 clinical studies will be needed for personalized medicine. This includes genetic, environmental, and medical history factors (Love et al., 2010; Schork, 2015).

Nanotechnology enables personalized oncology, in which cancer therapy and diagnosis are tailored to each patient’s tumor molecular profile, and predictive oncology, in which genetic and/or molecular markers predict development and progression of disease and clinical outcomes. The National Cancer Institute in the US has recently allocated funds to eight national Centers of Cancer Nanotechnology Excellence due to its potential impact on cancer research (Misra et al., 2010). Nanoparticles have a bright future as a new generation of cancer therapeutics because they offer the opportunity for the design and tuning of properties that other types of therapeutic drugs do not. There are still many challenges for the clinical development of nanoformulations, but as sufficient availability of clinical data is obtained, nanotechnology will lead to the rational design of optimized nanosystems with improved efficacy, selectivity, and safety. But our current understanding of nanocarrier safety is inadequate. Health risks associated with various nanosystems should be documented, and the pharmacokinetic behavior of various nanoparticles must be thoroughly studied. Preliminary and complementary animal studies should be conducted to identify nanoparticle risks, with a focus on elimination processes. Environmental and health effects of manufacturing these particles have received little attention. Given the many potential uses of nanoparticles in health, especially cancer research, the government must develop safety guidelines.

## 5. Conclusion

The precise delivery of therapeutic cargoes into ovarian tumor cells through novel engineered carriers from nano scaffolds has been widely explored in recent years. In the diagnosis and treatment of ovarian cancer, the chemotherapeutic drugs’ adverse effects were significantly curtailed, the site-specific delivery was augmented and solubility issue of hydrophobic cargoes was resolved using numerous nanotechnology-based carrier systems. In ovarian cancer, nanotechnology from nanoscience domain addresses the site-specific delivery and provides new-enhanced and targeted therapy possibilities. Several formulations show interesting results but only a few will make their way to clinical trials and clinics because the progress of most of them is halted in pre-clinical stages due to a number of factors. Regarding clinical translation, each nanoformulation has particular challenges related to either biological, study-design, or technology related. In addition, the toxicity, biotransformation and excretion of nanocarriers-based systems for ovarian cancer will also be a challenging aspect. Therefore, in the design stages, such considerations should be kept in mind for developing biocompatible and nontoxic carrier systems. Numerous nanotechnology-based biosensors have been proposed and tested in recent years for the diagnosis of ovarian cancer. The associated concerns with ovarian cancer diagnosis and treatment could be resolved through these nanomaterials based biosensors due to their high sensitivity and selectivity. However, the development of nanosensors is a time-consuming and complex process and thus often fails in the critical evaluation of biomarkers. Importantly, in the identification of ovarian cancer biomarkers, the demand of portable nanosensors that could be used to rescue subjects outside the clinical settings is of much value. In the scenario of ovarian cancer diagnosis, microfluidic and paper-based nanosensors fabrication offers feasible prospect for biosensors’ commercialization. In a nutshell, in the near future thrilling developments are required for sensing procedure scale-down using nanotechnology platform that hopefully would enable the patients to check their health easily and indeed would open new avenues in the diagnosis and treatment of ovarian cancer.
